# Cultivating the future leaders of chemical biology

**DOI:** 10.1039/d4cb90055c

**Published:** 2024-11-22

**Authors:** Anna Rulka, Elizabeth Adams, Akane Kawamura, Stephen Wallace

**Affiliations:** a Royal Society of Chemistry UK; b Scafell Coaching UK; c Chemistry – School of Natural and Environmental Sciences, Newcastle University Bedson Building Newcastle NE1 7RU UK akane.kawamura@newcastle.ac.uk; d Institute of Quantitative Biology, Biochemistry and Biotechnology, School of Biological Sciences, University of Edinburgh Edinburgh EH9 3FF UK stephen.wallace@ed.ac.uk

## Abstract

The inaugural RSC Chemistry Biology Interface Community Leadership Retreat for Early Career Researchers aimed to provide a unique combination of research leadership training, discussion and collaboration opportunities to emerging chemical biologists from the UK and Europe. This article outlines the ethos and reports the outcomes from this new event for the Royal Society of Chemistry and future plans to establish this event as an ongoing feature in the chemical biology conference landscape.

## Abstract 2

The inaugural RSC Chemistry Biology Interface Community Leadership Retreat for Early Career Researchers provides unique and immersive training in diverse research skills essential to emerging future leaders in chemical biology.

## Graphical abstract

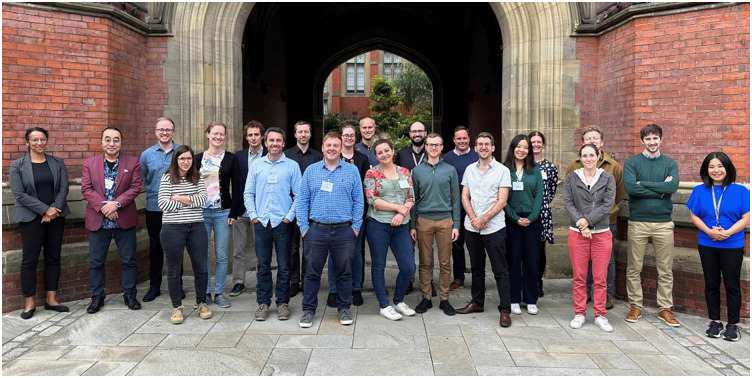

In a landscape where emerging scientists are expected to not only produce cutting-edge research but also lead innovative teams, the need for structured leadership development has never been more critical. The RSC Chemistry Biology Interface Community (RSC-CBIC) Leadership Retreat for Early Career Researchers (ECRs), held in September 2023 at the University of Newcastle, is a response to this evolving need. Co-chaired by Professors Akane Kawamura, Newcastle University and Stephen Wallace, University of Edinburgh, this event offered participants (Fig. 1) an immersive experience in leadership, innovation, and collaboration.

**Fig. 1:**
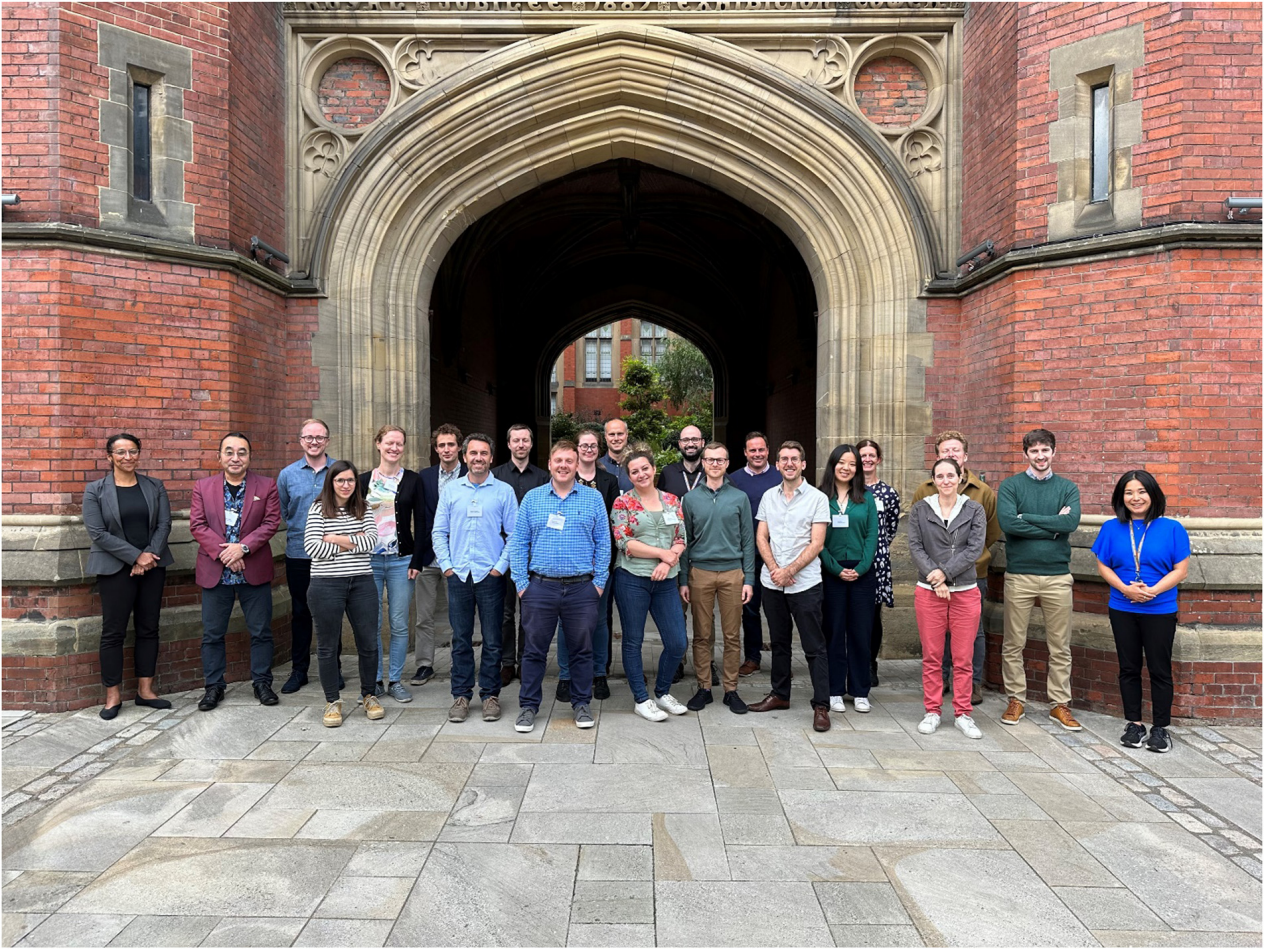
The 2023 cohort attending the 1st RSC-CBIC Leadership Retreat for Early Career Researchers.

While most scientific gatherings focus solely on technical knowledge and research outcomes, the RSC retreat focused on what it means to lead in academia today. Across three days, participants explored the complexities of managing a lab, building strong research teams, and navigating the mental health challenges that can arise in high-pressure academic settings. With sessions that combined theory and practical guidance, this retreat wasn’t just about science – it was about shaping the leaders who will guide the next generation of chemical biologists.

## A new focus on leadership

**Fig. 2:**
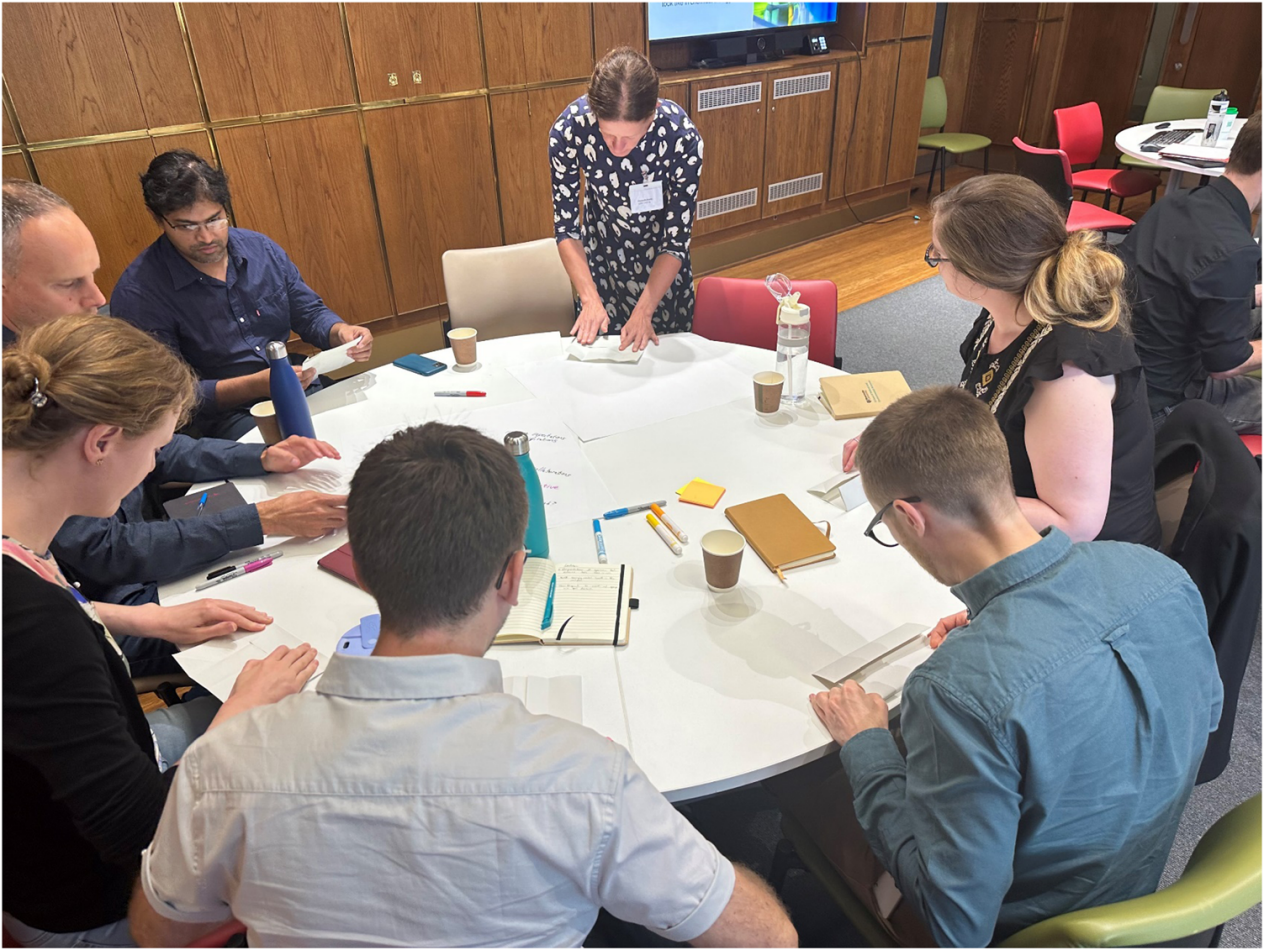
The leadership development workshop designed by Dr Elizabeth Adams from Scafell Coaching focussed on developing effective and supportive team environments.

Early-career researchers, particularly those establishing their own labs, are often thrown into leadership roles without formal training or support. The organizers addressed this challenge by focusing a major part of the retreat on leadership development, including workshops on lab management led by Dr Elizabeth Adams from Scafell Coaching, which provided attendees with tools that extend beyond the science bench (Fig. 2). For many ECRs, the transition from researcher to leader is challenging, from recruiting the right team to managing conflicts, and developing a supportive environment.

The discussions around creating positive lab cultures were especially relevant. Attendees were encouraged to think deeply about their leadership styles, the mental health of their teams, and how to sustain motivation in the long term. Strategies such as hosting lab retreats and setting clear expectations resonated with participants who are navigating the early stages of their independent careers. These are the kinds of insights that are rarely discussed in formal academic training but are crucial to the success of any research group.

## Honest reflections and shared experiences

One of the most refreshing aspects of the retreat was its honest approach to leadership. In a world where academic success is often measured by the number of publications and grant awards, this event provided a space for real, unfiltered conversations. From discussing career regrets to openly addressing failures, participants were able to share their journeys in a way that encouraged vulnerability and trust.

Professor Hiroaki Suga’s talk on his career path, including his successes and missteps, offered attendees a rare glimpse into the realities of an academic career. His openness about the complexities of commercializing research and balancing personal and professional priorities allowed participants to see beyond the traditional metrics of success. It was a reminder that research leadership is as much about resilience as it is about innovation.

## The essential role of innovation and collaboration

The retreat also emphasized the importance of collaboration in driving innovation. In a series of five-minute “chalk-talk” presentations, attendees pitched new research ideas and received immediate feedback from their peers. This exercise not only fostered creativity but also built a sense of community, encouraging participants to form collaborations that may shape the future of their research.

In a field as interdisciplinary as chemical biology, where the lines between academia and industry are increasingly blurred, networking is key to long-term success. The retreat provided a ground for these connections to grow, with participants leaving not just with new knowledge but with new professional relationships that could influence their careers for years to come.

## Leadership beyond the lab

**Fig. 3:**
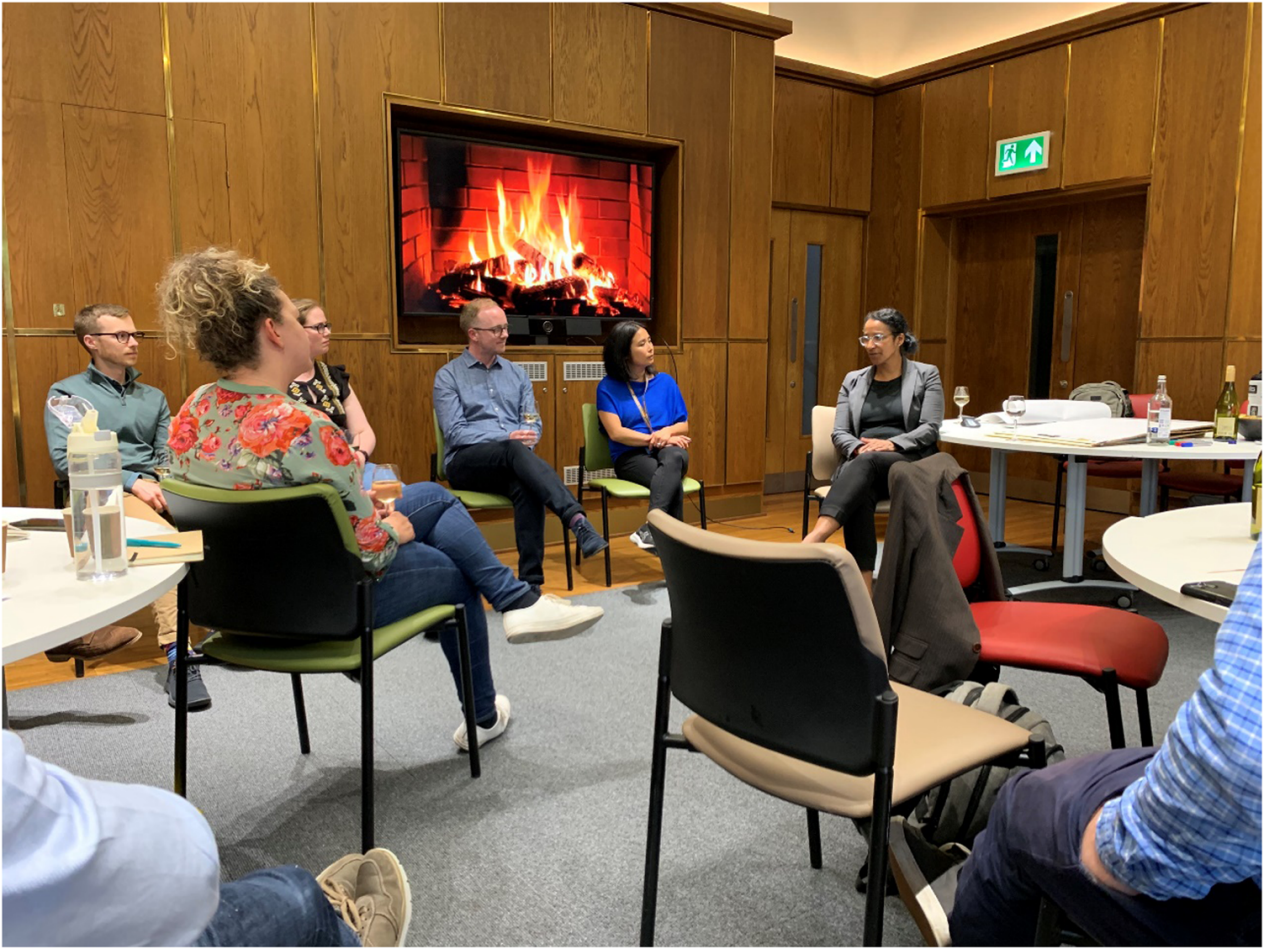
An evening Fireside Chat with plenary speaker Dr Lydia Meyer-Turkson around strategic aspects of research leadership in the biopharma industry and the importance of diversity and inclusion in leadership decision making.

Perhaps the most powerful message of the retreat was that leadership in science goes beyond managing people in a lab. Dr Lydia Meyer-Turkson’s plenary talk (Fig. 3) offered a deep dive into the broader strategic aspects of leadership, drawing from her experience in the biopharma industry. She highlighted the importance of diversity and inclusion in decision-making, a critical issue in both academic and corporate settings.

Dr Meyer-Turkson’s reflections on work-life balance, career progression, and talent retention resonated with many participants who are dealing with these issues in their own careers. Her openness about her experiences as a Black woman in academia and industry added a vital layer to the conversation on how to create inclusive, supportive environments in science.

## A blueprint for future success

As the inaugural event of its kind, the RSC Leadership Retreat for ECRs set a powerful precedent for future leadership development in chemical biology. It offered participants not just the tools to lead but the confidence to embrace the challenges of leadership with honesty and resilience. As early-career researchers return to their respective labs, they do so armed with practical strategies, a supportive network, and a renewed sense of purpose.

In a field where the pressures to publish, secure funding, and lead teams can feel overwhelming, events like this are not just beneficial—they are essential. By equipping the next generation of researchers with the skills they need to lead with empathy, innovation, and integrity, the RSC is helping to shape the future of chemical biology. This is only the beginning, and as this biennial event grows, so will the community of scientific leaders it provides support for.

Acknowledgments are given to those who organized and supported the event, particularly Professor Ali Tavassoli, Dr Anne Horan, and the tireless efforts of Rose Bunker and Katy Sawyer from the University of Newcastle, as their collaborative effort made this retreat a definite success.

